# Diversity and structural‐functional insights of alpha‐solenoid proteins

**DOI:** 10.1002/pro.5189

**Published:** 2024-10-28

**Authors:** Paula Nazarena Arrías, Zarifa Osmanli, Estefanía Peralta, Patricio Manuel Chinestrad, Alexander Miguel Monzon, Silvio C. E. Tosatto

**Affiliations:** ^1^ Department of Biomedical Sciences University of Padova Padova Italy; ^2^ Department of Protein Science KTH Royal Institute of Technology Stockholm Sweden; ^3^ Laboratorio de Investigación y Desarrollo de Bioactivos (LIDeB), Departamento de Ciencias Biológicas, Facultad de Ciencias Exactas Universidad Nacional de La Plata La Plata Buenos Aires Argentina; ^4^ Laboratorio de Farmacología Molecular Universidad Nacional de Quilmes Bernal Buenos Aires Argentina; ^5^ Department of Information Engineering University of Padova Padova Italy; ^6^ Institute of Biomembranes, Bioenergetics and Molecular Biotechnologies National Research Council (CNR‐IBIOM) Bari Italy

**Keywords:** alpha‐solenoids, ankyrins, armadillo repeats, HEAT, structured tandem repeats, tandem repeat classification, TPR repeats

## Abstract

Alpha‐solenoids are a significant and diverse subset of structured tandem repeat proteins (STRPs) that are important in various domains of life. This review examines their structural and functional diversity and highlights their role in critical cellular processes such as signaling, apoptosis, and transcriptional regulation. Alpha‐solenoids can be classified into three geometric folds: low curvature, high curvature, and corkscrew, as well as eight subfolds: ankyrin repeats; Huntingtin, elongation factor 3, protein phosphatase 2A, and target of rapamycin; armadillo repeats; tetratricopeptide repeats; pentatricopeptide repeats; Pumilio repeats; transcription activator‐like; and Sel‐1 and Sel‐1‐like repeats. These subfolds represent distinct protein families with unique structural properties and functions, highlighting the versatility of alpha‐solenoids. The review also discusses their association with disease, highlighting their potential as therapeutic targets and their role in protein design. Advances in state‐of‐the‐art structure prediction methods provide new opportunities and challenges in the functional characterization and classification of this kind of fold, emphasizing the need for continued development of methods for their identification and proper data curation and deposition in the main databases.

## 1 INTRODUCTION

Tandem repeat proteins (TRPs) are defined by the presence of periodically repeated amino acid sequences (Andrade, Perez‐Iratxeta, & Ponting, 2001; Heringa, 1998; Kajava, 2012) and are the second most abundant class of proteins involved in protein–protein binding after immunoglobulins (Li et al., 2006). They have been reported to be ubiquitous in genomes, with at least 14% of protein‐coding sequences containing repeats (Marcotte et al., 1999). Recently, a specific subset of TRPs has been defined: “structured tandem repeat proteins” or STRPs, which are mainly characterized by their defined modular 3D structure, among other characteristics (Monzon et al., 2023). The conformational space of STRPs is dominated by elongated (class III) and closed structures (class IV), according to Kajava's classification (Kajava, 2012). Class III is mostly composed of solenoid structures characterized by repeating structured units of 5–40 residues, forming elongated folds in contrast to globular proteins. The so‐called “alpha‐solenoids,” mainly composed of alpha‐helices, are the most common (Kajava, 2001; Kobe & Kajava, 2000) and have been intensively studied in the last two decades. Solenoidal folds, in general, can also be functionally characterized by their structural properties, such as curvature, handedness, and twist, which contribute to their hierarchical classification (Kobe & Kajava, 2000).

**FIGURE 1:**
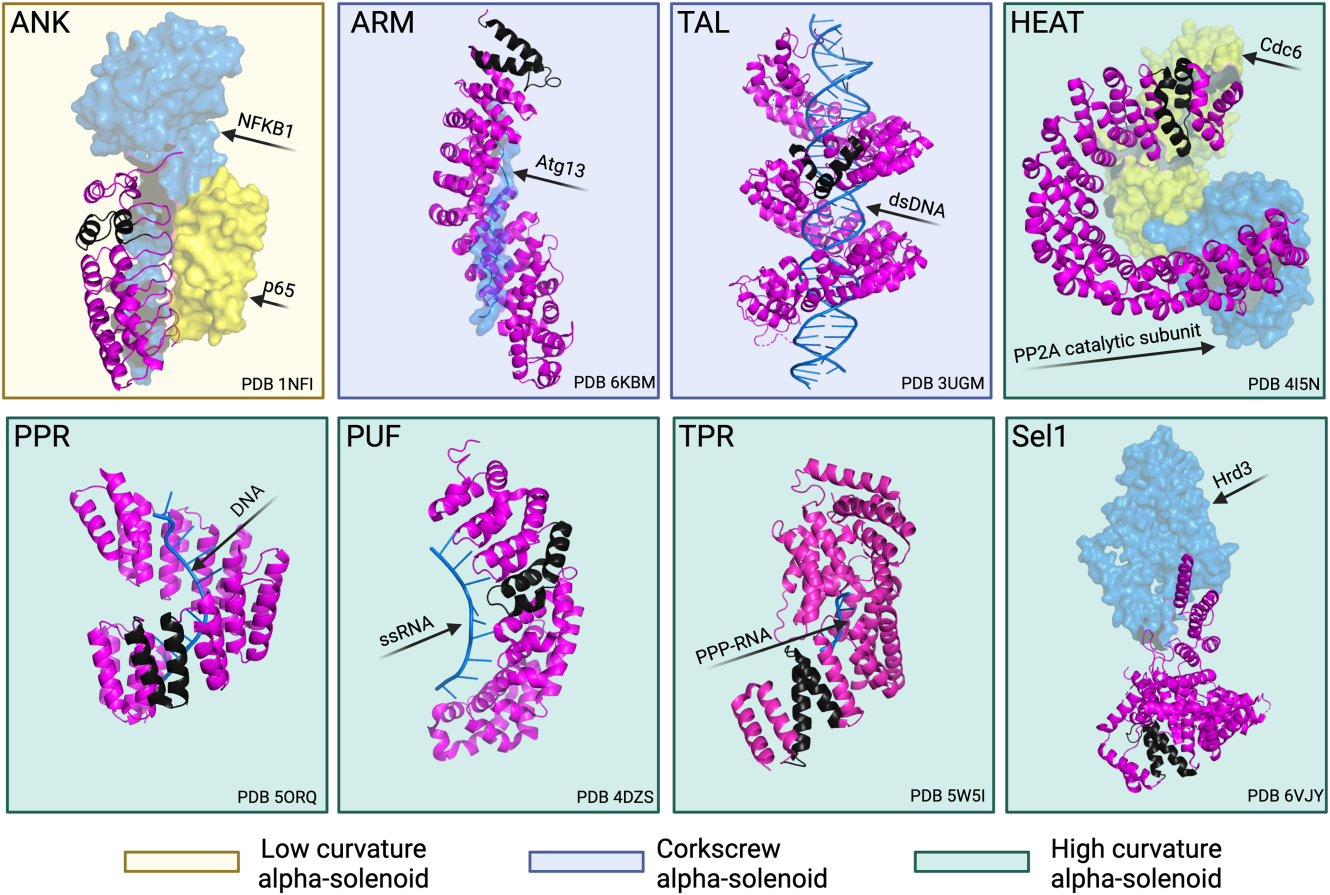
Representative structures of the main eight subfolds of alpha‐solenoid proteins under three folds and their interaction with proteins/peptides/nucleic acids. Repeat‐containing chain is in magenta color and a typical unit is highlighted in dark gray. The interacting proteins/peptides are shown as surfaces. ANK: *Homo sapiens* nuclear factor kappa‐light‐chain‐enhancer of activated B cells (NF‐kappa‐B) inhibitor alpha in complex with transcription factor p65 and NFKB1 (PDB: 1NFI), ARM: *Saccharomyces cerevisiae* Vac8 in complex with Atg13 (PDB: 6KBM), TAL: *Xanthomonas oryzae* TAL effector protein pthXo1 bounds to dsDNA (PDB: 3UGM), HEAT: *H. sapiens* serine/threonine‐protein phosphatase 2A in complex with Cdc6 and PP2A catalytic subunit (PDB: 4I5N), PPR: designed cPPR‐Telo1 in complex with DNA (PDB: 5ORQ), PUF: *S. cerevisiae* Puf4p bound to ssRNA (PDB: 4DZS), TPR: *H. sapiens* IFIT1 with PPP‐RNA (PDB: 5W5I), Sel1: *S. cerevisiae* Hrd1/Hrd3 monomer where Sel1‐like Hrd1 interacts with Hrd3 (PDB: 6VJY).

RepeatsDB (Paladin et al., 2021) is a comprehensive, manually curated database focused on the identification, classification, and analysis of STRPs. In its efforts to classify and describe alpha‐solenoids, RepeatsDB categorizes them based on their class and topology as elongated repeats/alpha‐solenoids, as well as on their fold structural properties, such as low and high curvature, and corkscrew. Moreover, alpha‐solenoids are defined not only by their structural differences but also by the presence of different sequence motifs, making it possible to identify diverse subfolds (Figure 1). Here we review the main types of alpha‐solenoidal STRPs subfolds, namely ankyrins (ANK); Huntingtin, elongation factor 3 (EF3), protein phosphatase 2A (PP2A), and target of rapamycin (TOR) (HEAT); armadillo (ARM); tetratricopeptide (TPR); pentatricopeptide (PPR); Pumilio (PUF) repeats; TAL effector repeat (TAL); and Sel‐1 and Sel1‐like repeats (SLRs). We provide a brief description of their structure and sequence patterns as well as functionally relevant examples for each type of repeat. We also highlight noteworthy cases of designed alpha‐solenoid proteins that are not naturally occurring but are of particular interest to researchers.

## 2 ALPHA‐SOLENOIDS SUBFOLDS

**FIGURE 2:**
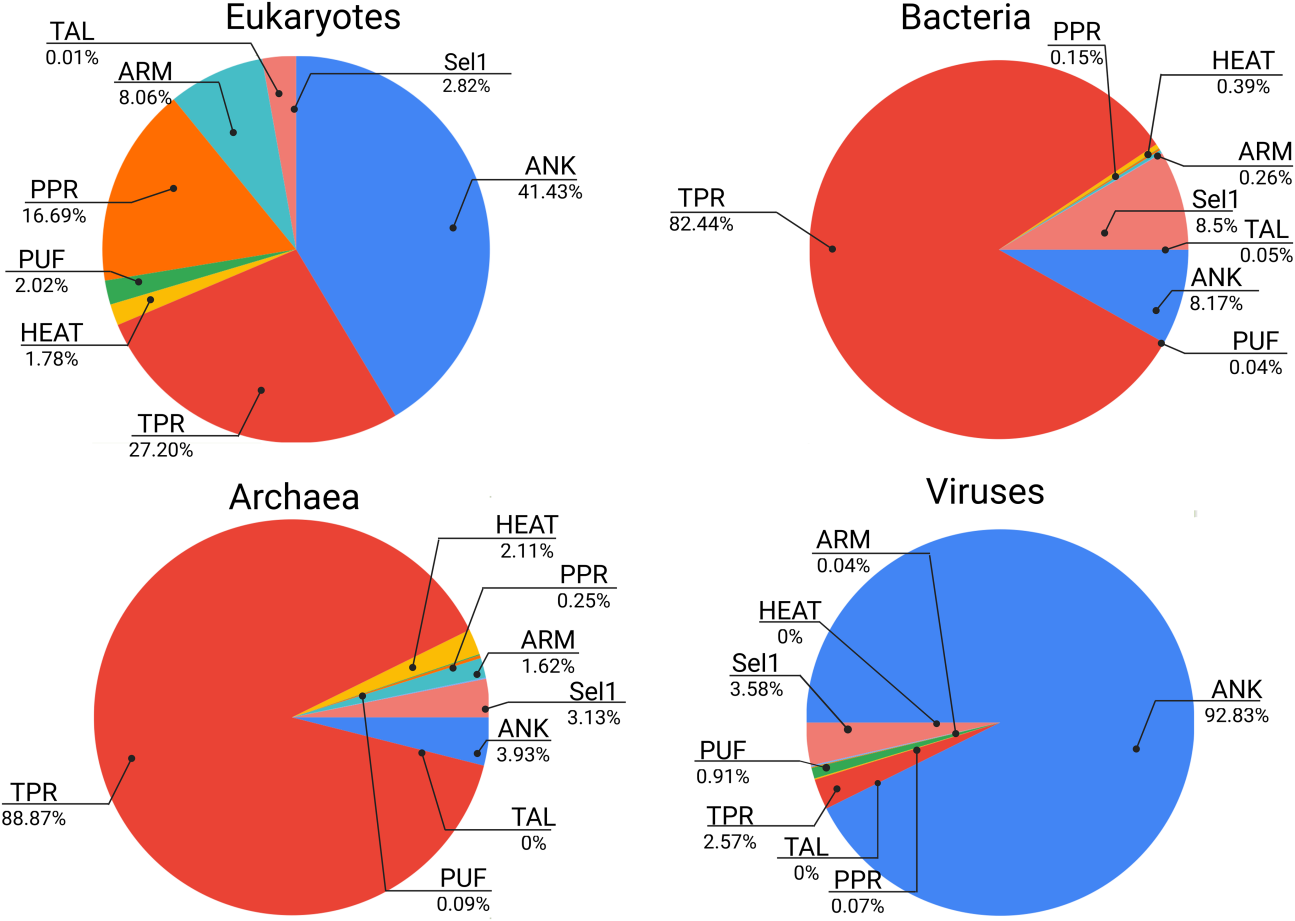
Proportion of alpha‐solenoid proteins by subfolds in the tree of life. Proportions were obtained by dividing the total number of alpha‐solenoid proteins of each subfold by the total number of all alpha‐solenoid proteins. ANK, ankyrin repeats; ARM, armadillo repeats; HEAT, Huntingtin; Sel1, Sel‐1‐like repeats; PPR, pentatricopeptide repeats; PUF, Pumilio repeats; TAL, transcription activator‐like; TPR, tetratricopeptide repeats.

The distribution of subfolds across the tree of life varies significantly in some particular cases (Figure 2 and Table 1). ANK and TPR are prominent across all domains but with varying proportions. Eukaryotes have a more diverse representation of subfolds, while Bacteria and Archaea are heavily dominated by TPR. Viruses show a strong preference for ANK, mostly due to their presence in poxviruses (Herbert et al., 2015). However, the presence of alpha‐solenoid subfolds in viruses is highly depleted compared to the other kingdoms. On the contrary, Eukaryotes display the highest diversity in alpha‐solenoid subfolds, which aligns with their complex cellular machinery and functions. The diversity of alpha‐solenoids is also reflected in their subcellular localization and associated functions, including cell cycle control, cell signaling, apoptosis, transcriptional regulation, cellular scaffolding, development, and differentiation, among others (Li et al., 2006) (Table S1).

**TABLE 1:** Subfolds of the alpha‐solenoid proteins and their distribution across the tree of life (UniProtKB accessed on June 17, 2024). The highest absolute number of proteins for each alpha‐solenoid subfold among the tree of life is underlined.

Abbreviation	Name	Number of repeat proteins (UniProtKB)				
		Eukaryotes	Bacteria	Archaea	Viruses	Total
ANK	Ankyrin	565,710	71,112	786	6853	637,608
ARM	Armadillo	110,109	2261	323	3	112,718
TAL	TAL effector repeat	2	407	N/A	N/A	409
HEAT	HEAT	24,349	3378	422	0	28,202
PPR	Pentatricopeptide	227,968	1288	49	5	229,305
PUF	Pumilio	27,534	382	18	67	28,015
TPR	Tetratricopeptide	371,396	717,207	17,776	190	1,114,754
Sel1	Sel1‐like repeats	38,494	73,919	626	264	114,318

## 3 ANKYRIN REPEATS

**FIGURE 3:**
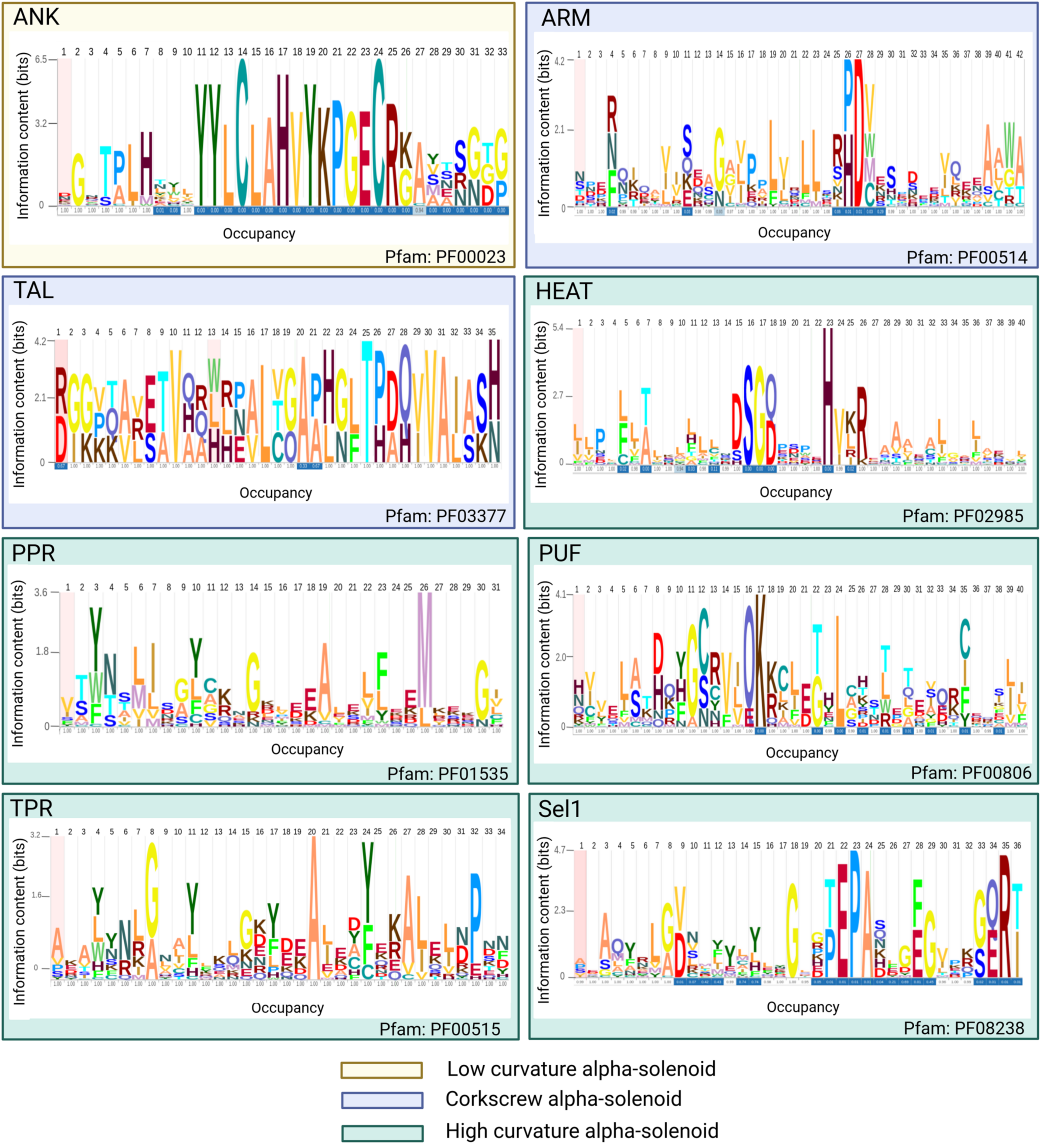
Sequence logos of main subfolds of alpha‐solenoid proteins. One representative Pfam family per each subfold is considered. The sequence logos were made by the Skylign tool (Wheeler et al., 2014) with default parameters using seed Pfam alignment as an input. Occupancy is the probability of observing a letter at position *k*, and every amino acid has a unique color by default parameters. The length of the sequence logos is modified to fit into one repeat unit length of the specific Pfam superfamily. ANK, ankyrin repeats; ARM, armadillo repeats; HEAT, Huntingtin; Sel1, Sel‐1‐like repeats; PPR, pentatricopeptide repeats; PUF, Pumilio repeats; TAL, transcription activator‐like; TPR, tetratricopeptide repeats.

ANKs repeats are one of the most common protein sequence motifs and, as such, are involved in a myriad of key cellular processes (Figure 1; Tables 1 and S1). Each repeat has a length between 30 and 34 amino acids and presents a helix‐turn‐helix conformation (Li et al., 2006). Structurally, ANK regions consist of repeats of antiparallel alpha‐helices stacked side by side and connected by beta‐hairpin motifs, where the extended beta‐sheets project away from the helical pairs almost at right angles to them, resulting in a characteristic L‐shaped cross‐section (Figure 1). Specific residues are preserved in key consensus positions, which are essential for a correct conformation: Gly in positions 4, 15, and 27 of the repeat unit, the Thr‐Pro‐Leu‐His9 tetrapeptide (TPLH) motif (Figure 3). While the interactions between these conserved residues result in the non‐globular conformation of the domain, these proteins remain functional both within the cell and in the extracellular space (Michaely, 2002; Sedgwick & Smerdon, 1999). Generally, the structure is mainly stabilized by intra‐ and inter‐repeat hydrophobic interactions and hydrogen bonds (Li et al., 2006). Lastly, a group of six non‐conserved residues on the surface of the protein drive peptide recognition, providing each protein with specific binding partners (Magliery & Regan, 2005).

Protein sequences of ANK repeats are highly divergent, with sequence identity falling below 20% in certain cases. Additionally, it has been shown that internal repeats are better conserved than those at the N‐ and C‐terminal regions, with N‐terminal repeats being the least conserved (Herbert et al., 2015). Multiple insertions and deletions occurring in specific repeating units often play a role in protein–protein interactions (PPIs) (Utgés et al., 2021). These positions are typically enriched in highly frustrated interactions, with potential impact on the recognition process and folding dynamics (Sanches et al., 2022).

ANK are a family of scaffold proteins that mainly bind other proteins (Figure 1), making them central to several key cellular processes, including but not limited to, cell signaling, cytoskeleton integrity, inflammatory response, and different transport phenomena (Bennett & Chen, 2001; Mosavi et al., 2004; Sedgwick & Smerdon, 1999).

In mammals, there are three canonical ankyrin proteins: ankyrin‐1 (or erythrocyte ankyrin, also known as ankyrin‐R), ankyrin‐2 (or brain ankyrin, also known as ankyrin‐B), and ankyrin‐3 (or ankyrin‐G), which are encoded by genes *ANK1*, *ANK2*, and *ANK3*, respectively. The canonical isoform of *Homo sapiens* ankyrin‐1 has 23 repeats and is part of the ankyrin‐1 complex, which is involved in the stability and shape of the erythrocyte membrane (Vallese et al., 2022), while the canonical isoform of ankyrin‐2 has 24 repeats and is highly expressed in cardiac muscle, where it has an essential role in the localization and membrane stabilization of ion transporters and channels. Human ankyrin‐3 has 23 repeats, and it is specifically localized to the neuromuscular junction and nodes of Ranvier (Kordeli et al., 1995) via the interaction with SCHIP1 (Papandréou et al., 2015).

Genetic alterations such as point mutations, deletions, and insertions in the genes that encode ANK‐containing proteins result in dysfunctional proteins and subsequently lead to various pathologies, including cancer and neurological disorder. Some of these mutations are located within repetitive regions of ANK‐containing proteins. For example, familial melanoma‐associated mutations within ANK repeat regions in p16 interfere with the biological function of this molecule. Notably, residues D74 and R87 directly interact with cyclin‐dependent kinases and are conserved among INK4 family members and p16 orthologs (Li et al., 2006; Mosavi et al., 2004).

As stated before, ANK proteins are present in a wide range of organisms. For example, ankyrin repeat protein 2A (AKR2A) of *Arabidopsis thaliana*, which has four ankyrin repeats at the C‐terminal end, is involved in disease resistance and antioxidant metabolism (Yan et al., 2002), and also displays chaperone activities toward membrane proteins located in the chloroplast outer envelope membrane (OEM) (Bae et al., 2008). AKR2A is also involved in the flowering process via the interaction with the FLOWERING LOCUS T (FT) protein, as loss‐of‐function mutant plants for AKR2A present a delayed flowering phenotype (Tang et al., 2022). In *Triticum aestivum* (common wheat), the Tiller Number1 (TN1) gene encodes an ankyrin protein with a transmembrane domain. Mutations in a single residue of the third conserved ankyrin repeat in TN1 cause a decrease in tillers, which are key elements of the plant that directly contribute to crop yield (Dong et al., 2023).

Some ANK proteins are key for the pathogenesis of intracellular bacteria. For instance, *Legionella pneumophila* modulates the host's processes in order to establish the *Legionella*‐containing vacuole (LCV), where it replicates by employing multiple effector proteins. The protein RomA is one of these effectors; it contains six ANK repeats and is required for histone H3 binding and subsequent modification of the host's chromatin (Rolando et al., 2023). Another effector is the ankyrin‐containing GTPase LegA15, which hijacks the host vesicular transport by promoting Golgi fragmentation and co‐opting p115 of the host (Chen et al., 2022). This induction of the Golgi apparatus fragmentation is not exclusive to *Legionella* ankyrin proteins, as the ANK protein RARP2 of *Rickettsia rickettsii* has also been found to induce selective fragmentation of the trans‐Golgi network (Aistleitner et al., 2020). Another ANK protein from *Rickettsia* spp., RARP1, promotes entry, intracellular growth, and host‐cell invasion, presumably by interaction with multiple binding partners (Sanderlin et al., 2022).

Although ANK repeats are the most prevalent among eukaryotes, they are also predominant in viruses (Figure 2 and Table 1). The most notable instances of ANK proteins existing in viral genomes belong to poxviruses (Odon et al., 2018). Analyses show that the sequences of viral ANKs are divergent and support extensive gene duplication in chordopoxvirus (Sonnberg et al., 2011). For example, the 282E4 strain of the avipoxvirus, which infects several species of birds (Bolte et al., 1999) and has been extensively used for the development of recombinant vaccines, codes for 57 ankyrin repeat proteins (Deng et al., 2023). The function of these proteins has not been experimentally confirmed, but Kyoto Encyclopedia of Genes and Genomes (KEGG) and Gene Ontology (GO) analysis suggest that they might play a role in the transcription of viral genes (Deng et al., 2023).

The oncolytic virus myxoma virus (MV), which causes myxomatosis in rabbits, can infect and kill several human cancer cell lines by a mechanism that involves the protein M‐T5, which has seven ANK repeats (Wang et al., 2006). M‐T5 can bind and activate cellular Akt, and the susceptibility of the cell lines to MV infection is directly related to the level of Akt activation (Wang et al., 2006; Werden et al., 2007).

Although ANK proteins normally bind other proteins, it has been reported that they can also bind lipids. For example, the protein K1 of vaccinia virus, which is involved in the negative regulation of the NF‐κB signaling pathway, also promotes infection by binding and deforming the host's membrane through the basic amino acids of its ANK region (Kitamata et al., 2020).

ANK proteins are also found, albeit to a lesser extent, in archaea. For example, a thermophilic archaeal ANK protein, tANK of *Thermoplasma volcanium*, which has five repeats, has the particularity that presents three folding states (Löw et al., 2008).

In recent years, designed ankyrin repeats proteins (DARPins) have gained relevance (Forrer et al., 2003) because they constitute smaller, easier to produce, and high affinity binders that can be used instead of antibodies in multiple applications.

DARPins maintain the primary structure of ANK by retaining consensus sequences from the ANK family, preserving the most frequent residues for each canonical position but introducing changes into specific amino acids (Parra et al., 2015; Plückthun, 2015). These amino acids are located on the surface and involved in the binding interface, and thus are able to recognize a desired target with high specificity and affinity (Walser et al., 2022). Moreover, the introduction of a 19‐amino acid flexible loop into the β‐turn of DARPins allows for the selection of even higher affinity binding molecules (Schilling et al., 2014). Theoretical combinatorial libraries of DARPins can range from 5.2 × 10^15^ to 3.8 × 10^23^ for those containing two and three repeat modules (Plückthun, 2015), respectively. Highly diverse libraries can be produced easily and inexpensively by overlap‐extension polymerase chain reaction (OE‐PCR) using degenerate oligonucleotides (Morselli et al., 2024).

The broad applicability of DARPins is highlighted by the extensive amount of published work that employs them. One example of the use of DARPins for immuno‐oncology purposes is that of MP0250, a drug candidate that binds and inhibits vascular endothelial growth factor (VEGF) and hepatocyte growth factor (HGF), thus altering the tumor microenvironment (Baird et al., 2021). Antiviral drugs have also been developed based on this technology and tested in clinical trials; Ensovibep, an experimental therapy for COronaVIrus Disease of 2019 (COVID‐19) disease, was designed to engage the three units of the spike protein trimer of Severe Acute Respiratory Syndrome 2 (SARS‐CoV‐2) and inhibit ACE2 binding with high potency (Rothenberger et al., 2022). With the aim of improving these therapies, combined in silico and experimental studies have shown that certain residue positions are key to maintaining the general thermostability of these proteins, and DARPins introducing mutations in Asp17 present greater thermostability (Schilling et al., 2022).

## 4 ARMADILLO REPEATS

The ARM repeats, which owe their name to *Drosophila melanogaster*'s ARM protein where they were first identified (Nüsslein‐Volhard & Wieschaus, 1980; Riggleman et al., 1989), are motifs predominantly implicated in PPIs such as those mediating intracellular signaling, cell‐adhesion, and cytoskeletal regulation (Coates, 2003). A typical ARM repeat has a length of 42 residues and folds into three helices, denominated H1, H2, and H3, respectively. These helices are organized in a way in which H2 and H3 are packed antiparallel against each other and the shorter H1 is in a perpendicular position (Andrade, Petosa, et al., 2001) (Figures 1 and 3).

The ARM segment polarity protein of the fruit fly (UniprotKB acc. P18824), which is homologous to the highly evolutionary conserved β‐catenin (Valenta et al., 2012), contains 12 prototypical repeats and a truncated 13th repeat. It is involved in signal transduction of the Wnt signaling pathway, which plays essential roles in embryonic development as well as in adherens junctions of the central nervous system and epidermis (Cavallo et al., 1997; Loureiro & Peifer, 1998).

Cell‐adhesion is one of the well‐known biological functions performed by ARM repeat‐containing proteins. For example, in adherens junctions between cells, we find the junctional plaque protein plakoglobin. Plakoglobin, which in humans has 12 ARM repeats, is a key component of desmosomes and intermediate junctions and interacts with multiple proteins, including E‐cadherin (Aktary et al., 2017). ARM repeats are also present in a multitude of ubiquitin‐ligases, which are enzymes central to protein targeted‐ubiquitination and degradation. For example, the protein Ufd4 of *Saccharomyces cerevisiae* has four ARM repeats, which are essential for Ufd4 ubiquitination of its substrates (Ju et al., 2007).

In some cases, a sole ARM‐containing protein can participate in multiple biological processes. For example, Vac8 (UniProtKB acc. P39968) of *S. cerevisiae*, which has nine ARM repeats, is a key mediator for various cellular processes such as autophagy (Park et al., 2020), vacuole inheritance, and protein targeting (Wang et al., 2006), among others. Vac8 regulates these processes by interaction via its ARM repeats with different associated proteins (Kim et al., 2023), like Nvj1 (Jeong et al., 2017), Atg13 (Park et al., 2020), and Vac17 (Tang et al., 2006).

ARM repeats are also ubiquitous in plants, in which they participate not only in the processes mentioned above but also in growth, development, and the stress response (Mandal et al., 2018). For instance, the protein MLKS2 of *Zea mays*, which interacts via its N‐terminal ARM‐repeat region with F‐actin, is required for normal plant development and fertility (Gumber et al., 2019). In addition, both the ARM‐containing region as well as the C‐terminal non‐ARM region of EFR3 OF PLANT 3 (EFOP3) and EFR3 OF PLANT 4 (EFOP4), are required for pollen development in *A. thaliana* (Chen et al., 2022).

Several reports have linked ARM proteins in plants to the response to different stresses. In rice, Sharma and colleagues showed that a subset of ARM genes is differentially expressed in response to abiotic stress, that is, salt, cold, and dehydration (Sharma et al., 2014). Moreover, in *Arabidopsis*, the protein AtPUB2 is induced under oxidative stress and may contribute to the ability of plants to tolerate this type of stress, although the specific mechanism remains to be determined (Saini et al., 2023).

Although ARM repeats are also detected in the proteomes of bacteria and archaea, their numbers are significantly lower than in eukaryotes (Jernigan & Bordenstein, 2014) (Figure 2), and there are not many reports about them and their associated functions. The structure of the hypothetical protein TON1937 of the hyperthermophilic archaea *Thermococcus onnurineus* NA1, reveals a superhelix fold composed of seven alpha‐helical repeats, out of which only one, repeat 3, resembles a prototypical ARM (Jeong et al., 2013). In *Helicobacter pylori*, a pathogenic bacteria, the protein FliG, which contains several ARM repeats, forms an essential part of the flagellum. The ARM repeats of FliG are directly involved in the assembly of the MS‐C ring and responsible for the superhelical arrangement of the inner part of it (Xue et al., 2018). Another example of a bacterial protein with an ARM region is the protein PgDPP III of *Porphyromonas gingivalis*, a human pathogen responsible for periodontitis. PgDPP II is an atypical dipeptidyl‐peptidase (DPP) in which the ARM‐repeat domain, which is absent in typical DPPs, contributes to its enzymatic activity and influences its interdomain dynamics (Hromić‐Jahjefendić et al., 2017).

## 5 TRANSCRIPTION ACTIVATOR‐LIKE EFFECTORS

Transcription activator‐like effectors are proteins secreted by plant pathogens belonging to the β‐ and γ‐proteobacteria classes that bind DNA in a sequence‐specific way (Schornack et al., 2013) (Figure 1). The DNA‐binding domain of these proteins is composed of almost identical, 34 residue‐long repeats, each of which folds into a left‐handed, two‐helix bundle (Mak et al., 2012). The repeats associate with adjacent repeats to form a right‐handed superhelix that wraps around DNA. Sequentially, the repeats vary in two amino acid positions, 12 and 13, named “repeat‐variable diresidue” (RVD) (Boch & Bonas, 2010; Moscou & Bogdanove, 2009), which are responsible for the specific recognition of a DNA base (Figure 3).

Naturally, Transcription activator‐like effectors (TALEs) are virulence factors that support bacterial colonization of the plants by exerting their transcription factor activity (Doyle et al., 2013). Mainly, they are found in *Xanthomonas* spp. bacteria, which are responsible for the bacterial leaf blight and leaf streak diseases, which have devastating effects on rice production (Boch & Bonas, 2010; Verdier et al., 2012). TAL effector‐like proteins have also been found in fungal endosymbiotic bacteria and are key factors for the stress tolerance of the phytopathogenic fungal host (Carter et al., 2020) as well as for the maintenance of the endosymbiotic relationship (Richter et al., 2023) (Figure 2 and Table 1).

In addition to their importance for agriculture in terms of improving plant disease resistance, TALEs became interesting to researchers as genetic engineering tools. They have been employed to activate/repress expression of desired genes in different organisms, such as *Solanum lycopersicum*, *A. thaliana*, and also human cells (Cong et al., 2012; Geiβler et al., 2011; Morbitzer et al., 2010), and they have also been fused to nuclease domains like FokI to generate TALEN restriction enzymes. TALEN enzymes can be used to edit genomes by introducing double‐strand breaks in DNA. Although TALEN are not as popular as CRISPR, they have been utilized in vitro for the correction of mutations associated with different diseases, such as sickle cell disease (Moiani et al., 2024).

## 6 HEAT REPEATS

HEAT repeats owe their name to the proteins in which they were first identified; HEAT, eukaryotic translation elongation factor 3 (eEF3), regulatory A subunit of PP2A, and mechanistic target of rapamycin (mTOR) (Andrade & Bork, 1995; Andrade, Petosa, et al., 2001). One HEAT repeat is approximately 40 amino acids long, and its canonical structure consists of two helices (A and B) that form a helical hairpin (Figures 1 and 3). HEAT repeats stack against each other to form an elongated superhelix, in which the B helices form the concave surface important for interactions with other proteins (Cingolani et al., 1999). HEATs resemble ARM repeats; the helix A of HEAT corresponds to helices H1 and H2 of ARM, and helix B to ARM's helix H3. The similarity between these two types of repeats is suggested to be a consequence of convergent evolution (Andrade, Petosa, et al., 2001; Jernigan & Bordenstein, 2014). Later, in a separate study, analysis of mathematically defined repetitive short fragments of the protein sequences supported the structural homology between those two subfolds (Turjanski et al., 2016). As is also the case for ARM repeats, HEAT repeats are found in eukaryotic organisms as well as in bacteria and archaea. However, no viral proteins have been annotated as having HEAT repeats (Figure 2 and Table 1).

HEAT repeats are involved in signaling, cellular transport, and protein synthesis, among other processes. The different interactions that this type of repeat can carry out are a direct consequence of their structural plasticity, which allows HEAT repeats to adopt multiple tertiary conformations. The highly flexible and elastic conformational changes that occur in these repetitions are due to their characteristic hydrophobic core, which allows them to bind to specific partners as well as respond to intracellular environments (Kappel et al., 2010; Yoshimura & Hirano, 2016).

Huntingtin (HTT) is a 348‐kDa protein that is conserved from flies to mammals. Mutant variants (mHTT) generated by expansion of CAG repeats in exon 1 of the HTT gene located on chromosome 4 are the cause of Huntington's disease (HD) (Potkin & Potkin, 2018; Ross et al., 2014). mHTT protein can form β sheets and abnormal protein aggregates, and is involved in neurotoxicity and, subsequently, brain atrophy (Harper et al., 2005; Macdonald, 1993). A myriad of different interactors have been proposed for HTT. HAP40 has been experimentally validated as a HTT interactor, and this interaction has been shown to be conserved throughout evolution, although the specific function of the interaction remains unclear (Seefelder et al., 2020). Structural studies of the HTT‐HAP40 complex show that the HEAT domains of HTT wrap around HAP40, and biophysical characterization shows that the HAP40‐bound HTT is less prone to aggregation and presents greater structural stability, suggesting that HAP40 may stabilize HTT (Guo et al., 2018). The involvement of the HEAT repeats of HTT in the pathogenesis of HD, if any, has not been described. A recent study employing chimeric constructs of both wild‐type and pathogenic lengths of the exon 1 region of HTT and the HEAT region of PR65/A showed that both lengths of the exon 1 polyQ tract disturb the structure of PR65/A HEAT domain to the same extent. These results suggest that the interaction between the exon 1 polyQ tract and the HEAT repeats of HTT might play a role in both the physiological function of HTT as well as in the pathogenesis of HD, but further studies remain to be conducted (Zhang et al., 2023).

Eukaryotic eEF3 is encoded by YEF3 gene and has been isolated in different fungal species such as *S. cerevisiae*, *Candida albicans*, *Candida guillermondii*, and *Pneumocystis carinii*. eEF3 has ATPase and GTPase activities, in addition to being involved in EF‐Iα‐dependent binding of aminoacyl‐tRNA by the ribosome (Belfield et al., 1995; Sandbaken et al., 1990).

In eukaryotic cells, PP2A holoenzyme consists of a heterodimeric core enzyme composed of a scaffolding protein called subunit A and a catalytic subunit called subunit C (Xu et al., 2006). Subunit A has 15 HEAT repeats that are arranged in a particular L‐shaped fashion (Hemmings et al., 1990), which is necessary for its function: phosphorylation and dephosphorylation of signaling factors (Cho & Xu, 2007; Grinthal et al., 2010). Finally, the last protein responsible for HEAT's acronym is mTOR protein kinase. This protein is an essential component of two multiprotein complexes called MTORC1 and MTORC2, and contains 32 HEAT repeats (Chao & Avruch, 2019). This kinase is involved in both protein synthesis and autophagy, and it has been observed that deregulated mTOR signaling is directly related to the progression of cancer and diabetes (Saxton & Sabatini, 2017).

Tor1 kinase in *C. albicans*, has eight N‐terminal HEAT repeats in its protein–protein interaction domain. This less conserved region is important for *C. albicans* response to oxidative stress, cellular respiration, and hyphal growth. Furthermore, this site of the Tor1 protein is a therapeutic target because small drug‐type molecules that inhibit PPIs could be possible selective inhibitors of Tor1 kinase for fungi, thus being able to differentiate from human cells, a problem which is found in treatment with rapamycin (Qi et al., 2022).

HEATR1, a protein that contains a HEAT domain at C‐terminus, has been a target of extensive studies in recent years due to its involvement in different types of cancers and its potential as a therapeutic target. For example, in oral squamous cell carcinoma (OSCC), HEATR1 acts as a binding factor for the Pontin/Reptin complex, which is overexpressed in tumor cells (Nakamura et al., 2021), while in pancreatic cancer HEATR1 inhibits the activity of the Nrf2 factor that participates in the protection of cells from oxidative stress (Zhou et al., 2020). In the case of hepatocellular carcinoma (HCC) HEATR1 is up‐regulated through the IGF1‐mTORC1‐SP1 axis. It promotes the growth of HCC by enhancing ribosome biogenesis (Yang et al., 2023).

HEATs are also found in bacteria; for example, in *Myxococcus xanthus*, Cpc7 is a HEAT‐containing protein formed by 27 alpha‐helices which interacts with single‐domain response regulator (SD‐RR) CheY7. This protein–protein interaction regulates development in response to stress (Darnell et al., 2014). Gram‐positive bacteria such as *Bacillus cereus* and *Streptococcus mutans*, as well as Gram‐negative bacteria such as *Pseudomonas fluorescens* have HEAT‐like repeat (HLR) proteins, which are alkyl‐DNA glycosylases involved in DNA repair. The structure of the members of this superfamily is formed by an N‐terminal α‐helical bundle (NTB) (αA′‐αC) and five HLR motifs (αD‐αE, αF‐αG, αH‐αI, αJ‐αK, and αL‐αM) which make up a C‐shaped structure (Shi et al., 2018). In *P. fluorescens*, AlkC excises 7‐methylguanine, 3‐methyladenine, 3‐methylguanine, 3mC, and 1mA, and its residues Glu121 and Glu156, which belong to the HLR domain, are involved in this function. Although the structures of AlkC and AlkD are similar, the strategy to repair damaged DNA is different; AlkD only disturbs the helical axis and bases stacking to excise alkylated base, while AlkC opens the minor groove of the DNA in the injury (Alseth et al., 2006; Shi et al., 2018). In *S. mutans*, AlkD2 has a weak binding to DNA due to the absence of the αB helix in its structure. This protein is involved in purine metabolism, binding to insulin‐like growth factor 2 (IGF2) messenger RNA (mRNA)‐binding protein (IMP) through the loop that surrounds the αA and αC helixes, and the N‐terminal of the αG helix (Mullins, Shi, Kotsch, & Eichman, 2015; Mullins, Shi, Parsons, et al., 2015).

## 7 PENTATRICOPEPTIDE REPEATS

PPR repeat proteins were discovered in the genome of *A. thaliana* (Aubourg et al., 2000), and they were initially thought to be unique to plants. However, they were soon found in multiple other organisms (Figure 2, Tables 1 and S1), although in very low numbers when compared to the hundreds that are found in terrestrial plants (O'Toole et al., 2008). Similarly to TPRs, which have been evolutionarily linked to (Barkan & Small, 2014), a canonical PPR is a 35 amino acids motif that forms a helix‐turn‐helix, which together with the adjacent repeats fold into a superhelix (Figures 1 and 3). PPR proteins can be divided into two classes: P‐type proteins, which are composed mainly of canonical PPR motifs, and PLS‐type proteins, which consist of triplets that include a canonical PPR motif, a longer motif denominated as L, and a shorter one known as S. In general, repeat unit length varies between 31 and 36 residues (Yin et al., 2013). The number of PPRs in a protein is variable and it can go from two PPRs to as many as 28 like in *A. thaliana* PGR3 protein (UniProtKB acc. Q9SZ52).

The main binding partner of PPRs is RNA and the binding process is done in a sequence‐specific matter (Barkan & Small, 2014), which is in contrast to the few TPRs that are able to bind RNA (which do so in a sequence‐independent manner). The way PPR proteins recognize their RNA partners is done modularly; amino acid positions 5 and 35 within each repeat are responsible for the recognition and interact directly with specific nucleotides (Yagi et al., 2013; Yin et al., 2013). RNA binding abilities of PPRs make them central to multiple processes regarding organellar RNA metabolism, such as transcription, RNA‐processing (including 5′‐tRNA and 3′‐tRNA processing), polyadenylation, stability, splicing, translation, and editing. For instance, the protein PPR756 of *Oryza sativa* (rice) acts as an editing factor required for the editing of mitochondrial genes essential for development (Zhang et al., 2020). The structure of PPR756 has not yet been experimentally determined, but PPRs have been sequentially detected, and the AlphaFold2 (Jumper et al., 2021) model (accessible with the UniProtKB acc. Q2QTL4) predicts the characteristic ɑ‐solenoid that is expected for PPR‐containing proteins (Jumper et al., 2021; Varadi et al., 2022). The editing function is not limited to mitochondrial PPRs modifying mtRNAs. For example, the product of the *GmPGL2* gene of *Glycine max* (soybean) participates in C‐to‐U RNA‐editing in chloroplasts (Feng et al., 2021).

PPR proteins can also play a role in RNA stabilization. In *A. thaliana*, the protein PPR287 is crucial for chloroplast function, presumably via the stabilization of chloroplast ribosomal RNAs (Page et al., 1987) (rRNAs). The protozoan *Trypanosoma brucei*, which causes acute trypanosomiasis in humans, codes for multiple PPR proteins. Among these, six of them, TbPPR2 to TbPPR7, are key for mitochondrial rRNA stabilization (Pusnik et al., 2007), and another one, TbPPR9, selectively stabilizes the mRNAs of both cytochrome oxidase subunits 1 and 2 (COX1 and COX2, respectively) (Pusnik & Schneider, 2012). The protein CCM1 of *S. cerevisiae* also stabilizes mitochondrial rRNA by binding to a specific AU‐rich motif of the ribosome small subunit (Piątkowski & Golik, 2020).

Another key process in which PPRs are involved is protein translation, particularly of organelle‐encoded genes. The Pet309 protein of *S. cerevisiae* activates translation of COX1 by binding to the 5′ untranslated region (UTR) of its mRNA (Manthey & McEwen, 1995; Zamudio‐Ochoa et al., 2014). Its human ortholog LRPPRC (also known as Lrp130) plays a similar role, and mutations in the gene cause the French Canadian variant of Leigh syndrome, a neurodegenerative disorder (Cooper et al., 2006; Mootha et al., 2003; Xu et al., 2004). Recently, a study identified two PPR proteins in *A. thaliana* missanotated as adenylyl cyclases, which are required for translation initiation specifically in mitochondria (Tran et al., 2023). These two proteins, denominated mTRAN1 (AT4G15640) and mTRAN2 (AT3G21465), are part of the small subunit of the ribosome. The authors demonstrated that mTRAN1 binds to specific AU‐rich motifs in the 5′‐UTRs of mitochondrial genes, giving insight into the mechanism by which plant mitoribosomes recognize mRNAs even though they lack bacterial‐like Shine‐Dalgarno sequences (Tran et al., 2023).

Group‐II introns in plant organelles are introns that do not have the ability to self‐splice and they require the assistance of proteins to do so (Lambowitz & Zimmerly, 2011). PPR‐containing proteins can promote group‐II intron splicing thanks to their RNA binding capability. For example, the protein OsPPR939 of *O. sativa* is required for the splicing of introns 1, 2, and 3 of the *nad5* transcript, and knockdown or deletion of OsPPR939 severely affects development (Zheng et al., 2021). Something similar happens in maize, where the protein PPR4 is a trans‐splicing factor of rsp12 (Schmitz‐Linneweber et al., 2006) and PPR5 promotes *trnG* splicing (Beick et al., 2008) and *rpl16* splicing (Rojas et al., 2018).

An interesting and recently described PPR protein is OsCPPR1. This rice protein has 16 PPR motifs and is essential for tapetal plastid development via regulation of *OsGLK1* transcript levels, and what makes OsCPPR1 unique is the fact that it is the first cytoplasmic PPR to be reported (Zheng et al., 2022).

It is worth mentioning that the characteristics of PPR proteins, such as their modularity and sequence‐specific RNA binding ability, have made them attractive as biotechnological tools. Specifically designed PPRs can be used for controlling the expression of specific genes in organelles (Rojas et al., 2019), controlling splicing (Yagi et al., 2022) and to edit RNA directly without the use of cofactors (Bernath‐Levin et al., 2022).

## 8 PUMILIO REPEATS

PUF repeats can be found in the evolutionary conserved PUF family of RNA‐binding proteins. Typically, each PUF‐containing protein has eight repeats of around 36 amino acids, although some proteins, such as Puf‐A of *H. sapiens* and Nop9 of *S. cerevisiae* have 11 and Pum23 of *A. thaliana* has 10 repeats (Qiu et al., 2014; Tam et al., 2010; Zhang et al., 2016) (Figure 3). Each repeat folds into three alpha‐helices, which align with the helices of the following repeat, forming a crescent shape (Edwards et al., 2001; Wang et al., 2001) (Figure 1). The repeats present a degenerated sequence pattern, but three amino acid positions in the middle are conserved (Wang et al., 2002). These conserved positions are important because they make up the RNA‐binding surface of the PUF proteins; each repeat interacts directly with a RNA base (Figure 3) via the 3‐conserved residues in a sequence‐specific way (Wang et al., 2002). However, PUFs can also bind non‐cognate sequences (Gupta et al., 2008; Miller et al., 2008).

As post‐transcriptional regulators, PUF proteins are involved in a plethora of biological functions. They are usually repressors of gene expression, normally by binding to the 3′‐UTR of mRNAs and subsequently downregulating them, although mRNA stabilizing effects have also been reported (Archer et al., 2009; Kaye et al., 2009; Naudin et al., 2017). The specific mechanisms by which PUF proteins exert their role are diverse. For instance, PUF, one of the canonical and founding members of the PUF family, is involved in *D. melanogaster*'s early embryos development by binding hunchback (hb) mRNA, producing its translational arrest via deadenylation (Barker et al., 1992; Murata & Wharton, 1995) (removal of the poly‐A tail), and it also represses translation of eIF4E in the neuromuscular junctions of fruit fly larvae, modulating synaptic function (Menon et al., 2004).

Human PUF proteins PUM1 and PUM2 are important repressors that lower the levels of their target mRNAs. The mechanism can be both deadenylation‐dependent, for example, by directly binding the CCR4‐NOT deadenylase complex (Enwerem et al., 2021; Van Etten et al., 2012), and deadenylation‐independent, by promoting accessibility to miRNAs (Galgano et al., 2008; Kedde et al., 2010). *H. sapiens* also possesses non‐canonical PUF proteins; Puf‐A (also known as PUM3) has 11 repeats and is highly expressed in primordial germ cells (Kuo et al., 2009). Although it can bind ds‐RNA and DNA without sequence specificity (Qiu et al., 2014), its biological function is not clear. Its ortholog in yeast, Puf6, with which it shares less than 25% sequence identity but a similar structure, is involved in ribosome biogenesis (Gerhardy et al., 2021; Li et al., 2009). A recent work from Cho and collaborators (Cho & Xu, 2007) reported that Puf‐A promotes non‐small cell lung cancer (NSCLC) progression by the interaction with nucleophosmin (NPM1) in the nucleolus. Up‐regulated Puf‐A is also involved in breast cancer progression (Fan et al., 2013). This involvement in cancer is not unheard for PUF proteins; canonical PUM1 and PUM2 are altered in 17 types of cancer tissues (Smialek et al., 2021), have been shown to sustain cell growth in acute myeloid leukemia by promoting the expression of FOXP1 (Naudin et al., 2017) and the protein Nop9 has been suggested as a prognostic marker for renal cancer (Silva et al., 2022).

Proteins containing PUF repeats have also been identified and studied in fungi. For instance, *S. cerevisiae* Puf3p regulates downregulation of mitochondrial biogenesis (García‐Rodríguez et al., 2007; Saint‐Georges et al., 2008), and the proteins Puf3 and Puf4 play multiple functions in *Schizosaccharomyces pombe*, such as protection from toxic iron concentrations by repression of Frp1 expression (Beaudoin et al., 2021) and regulation of PI4P5K by suppression of its3‐1 (Satoh et al., 2023). Nop9, a nucleolar PUF with 10 repeats, is required for 18S rRNA synthesis by binding its central pseudoknot region and possibly regulating its folding (Wang & Ye, 2017).

As a probable consequence of gene duplication in plants, *A. thaliana* codes for 25 PUF proteins. Some of them are involved in stem cell maintenance and differentiation (Francischini & Quaggio, 2009), while others are required for normal growth and development (Abbasi et al., 2011; Zhang & Muench, 2015). Moreover, some are involved in responses to both abiotic and biotic stress, like APUM5 of *Arabidopsis* (Huh & Paek, 2014).

As it is the case with PPRs, given the ability of PUF proteins to regulate gene expression in a sequence‐specific matter, designer PUF proteins are interesting to the scientific community as biotechnological tools. Different approaches can be employed to, for example, enhance and suppress translation of target mRNAs (Campbell et al., 2014; Choudhury et al., 2012). For instance, one could employ natural variants of PUF proteins and engineer them in order to repurpose their biological function (Bhat et al., 2019) or generate different repeats that recognize different RNA bases (Zhao et al., 2018; Zhou et al., 2021). Adamala et al. (2016) generated four different PUF modules, which they have called “Pumby” (PUF‐based assembly), each of which recognizes specifically one RNA base and that can be combined with each other in order to bind desired target RNAs.

## 9 TETRATRICOPEPTIDE REPEATS

Initially described in yeast (Hirano et al., 1990; Sikorski et al., 1990); but now found to be widespread across organisms. They are more prevalent in bacteria and archaea (Figure 2, Tables 1 and S1). Each TPR repeat is approximately 34 amino acids in length and folds into a helix‐turn‐helix motif (Figure 1), which packs against the adjacent repeats, forming solenoid structures. Although TPRs present a degenerate sequence pattern, a consensus sequence has been defined for a group of amino acids (D'Andrea, 2003; Lamb et al., 1995) (such as small or large hydrophobic). Certain TPRs have 42 residue repeat units and more than 90% of sequence identity found, like those identified in *Podospora anserina* (Lamb et al., 1995) (Figure 3). The number of TPRs per protein varies; for example, *H. sapiens* TPR repeats protein 21B (UniProtKB acc. Q7Z4L5) and TPR repeat protein 28 (UniProtKB acc. Q96AY4) have 19 and 28 repeats, respectively.

TPRs are a highly diverse fold involved in PPIs and protein–peptide interactions for which several different binding modes have been described (Perez‐Riba & Itzhaki, 2019). This diversity of TPRs, both in structure and binding modes, is reflected in their associated functions, as they have been implicated in a plethora of processes that require PPIs, such as cargo recognition by kinesins (Pernigo et al., 2013), cell division (Yu et al., 2003; Zhu et al., 2011), peroxisomal import (Harper et al., 2003), and protein translocation across membranes (Schlegel et al., 2007), to mention a few.

Among these processes, an interesting case is the role of TPR‐containing proteins in the immune responses of different organisms. For instance, the interferon‐induced proteins with tetratricopeptide repeats (IFITs), which belong to the interferon‐stimulated genes (ISGs), play a critical role in the defense against viral pathogens in both humans and mice (Fensterl & Sen, 2011; Sen & Sarkar, 2007). Four IFIT proteins have been described in *H. sapiens* (IFIT1, IFIT2, IFIT3, and IFIT5) and three in *Mus musculus* (IFIT1, IFIT2, and IFIT3), all of which are composed mostly of TPRs motifs. None of these proteins are expressed in basal conditions, but instead their levels increase rapidly as a response to type I interferons (IFN‐I) (Daffis et al., 2007) or by signals produced after PRRs‐mediated (pattern recognition receptors) recognition of pathogen‐associated molecular patterns (PAMPs) (Sarkar & Sen, 2004). The mechanisms by which these proteins exert their antiviral functions are different. In the case of both IFIT1 and IFIT2 of *H. sapiens* and *M. musculus*, it has been shown that they can inhibit viral protein translation by binding eIF3 (eukaryotic initiation factor 3) and preventing the formation of the pre‐initiation complex (Diamond & Farzan, 2013; Guo et al., 2000; Vladimer et al., 2014). Regarding human IFIT3, its interaction via one of its TPRs with the serine/threonine‐protein kinase TBK1 enhances the IFN‐I antiviral signaling pathway. This effect is attributed to IFIT3 acting as a “bridge” between TBK1 and the mitochondrial antiviral signaling protein (MAVS) (Liu et al., 2011). Interestingly, although IFIT5 has been shown to positively regulate NF‐κB signaling via its interaction with IκB kinase (IKK) and the transforming growth factor‐β‐activated kinase 1 (TAK1) (Zheng et al., 2015), its antiviral activity is associated with its ability to specifically bind single‐stranded 5′‐PPP‐RNA (Abbas et al., 2013; Feng et al., 2013) (non‐capped RNAs that are specific to viruses). The RNA‐binding ability of TPRs is not unique to IFIT5, as the product of the *bsr‐k1* gene of *Oryza sativa* (rice) is a TPR‐containing protein that binds mRNA of multiple genes (Zhou et al., 2018). This demonstrates that while TPRs typically engage in PPIs, they are also capable of binding to nucleic acids (Figure 1).

TPR‐containing proteins are also involved in the tolerance of plants to different types of stress. For example, in *S. lycopersicum* (tomato), the protein SlREC2 enhances the cold tolerance of plants by interaction with SlBCH1b (Zhang et al., 2023), while the proteins DJC31 and DJC62 are involved in the abiotic stress responses of *A. thaliana*, presumably through the interaction with HSP80.1 and HSP90.2 (Dittmer et al., 2021). TPR proteins in plants are also important for development; *Z. mays* SMALL KERNEL 11 (*smk11*) is essential for the assembly of cytochrome c oxidase complex (the mitochondrial respiratory chain's complex IV) via its interaction with other four proteins, and this function requires all TPRs, as truncated forms are not sufficient for correct assembly (Ren et al., 2023).

In contrast to their roles in the immune defense of eukaryotes, some bacterial‐encoded TPR proteins play key roles in pathogenesis. In the opportunistic pathogen *P. aeruginosa*, the gene *pscG* encodes for a 115‐residue TPR‐containing chaperone, which is essential for the assembly of the type‐III secretion system commonly known as the injectisome, and this chaperone activity is mediated by its TPR motifs (Plé et al., 2010). Other virulence‐related functions of bacterial TPR proteins include immune evasion. The serine/threonine‐protein kinase PknG of *Mycobacterium tuberculosis* inhibits phagosome‐lysosome fusion, enabling the pathogen's survival inside macrophages (Walburger et al., 2004), while the protein BB0238 of *Borrelia burgdorferi*, the causative agent of Lyme disease, is required for various PPIs indispensable for evading the host's early immune responses (Foor et al., 2023), and specifically, its TPR motifs are essential for BB0238 stability in the environment of the host.

Other worth mentioning examples of functional bacterial TPR proteins are those of magnetotactic bacteria such as *Magnetospirillum gryphiswaldense* and *Magnetospirillum magneticum*. Both organisms have a specialized organelle called the “magnetosome” which allows their navigation along Earth's geomagnetic fields. The magnetosome biosynthesis is highly complex, but the MamA TPR protein is essential for its assembly (Lohße et al., 2014; Zeytuni et al., 2011). MamA contains five TPR repeats, and it interacts both with itself (Zeytuni et al., 2011) as well as with Mms6, a membrane‐bound magnetosome protein (Nguyen et al., 2016) to form a scaffold inside the magnetosome. Although TPRs are also present in archaea (Kyrpides & Woese, 1998) not much is known about their functions in these organisms.

In the case of viruses, a TPR‐containing protein of *Acanthamoeba polyphaga* mimivirus R856 (UniProtKB acc. Q5UQQ7) has been reported to be involved in fiber‐formation. The specific role of R856 and the importance of its TPR domain have not been established, but gene silencing experiments result in viruses with shorter and abnormal fibers, which suggest that R856 is either an essential component of fibers or a key factor in their biosynthesis (Sobhy et al., 2015).

Recent works have described a small‐communication system in SPbeta phages that regulates the lysis‐to‐lysogeny transition (Erez et al., 2017; Gallego Del Sol et al., 2022; Guan et al., 2019; Stokar‐Avihail et al., 2019). The system is composed of three genes, one of which codes for a transcription factor called AimR. AimR, which presents nine degenerated‐TPRs that are directly responsible for binding another of the system's gene products, a short peptide called AimP. AimP binding impedes AimR's transcription factor activity, thus promoting lysogeny (Gallego Del Sol et al., 2022).

## 10 SEL‐1 AND SEL‐1‐LIKE REPEATS

The SLRs are related to TPRs but their consensus sequence, in which conserved Ala and Gly residues can be found, is different (Mittl & Schneider‐Brachert, 2007) (Figures 1 and 3). First identified in the gene *sel‐1* (suppressor‐enhancer of *lin* 1) of *Caenorhabditis elegans* (Grant & Greenwald, 1996), SLRs have a length between 36 and 44 residues and fold into a helix‐turn‐helix motif, which together with the neighboring repeats form superhelical structures (Figure 3). The repeat number in SLR‐proteins varies; for example, *H. sapiens*' eukaryotic elongation factor 2 kinase (eEF‐2K) contains four SLRs, while *Escherichia coli*'s secretory immunoglobulin A‐binding protein (EsiB) contains 11.

In terms of function, SLRs are involved in multiple processes such as signal transduction (Ji et al., 2023; Mittl & Schneider‐Brachert, 2007), endoplasmic reticulum protein quality control (Cattaneo et al., 2009; Jeong et al., 2017) assembly of mitochondrial respiratory chain complexes (Kozjak‐Pavlovic et al., 2014) and activation of the integrated stress response (Fessler et al., 2020; Guo et al., 2020), among others. In particular, mutations in SEL1L have been linked to progressive early‐onset cerebellar ataxia in dogs (Kyöstilä et al., 2012) and to neurodevelopmental disorders in humans (Weis et al., 2024).

SLRs have also been identified in bacteria and have been implicated in several processes such as flagellar biogenesis (Sakuma et al., 2019), antibiotic resistance (Lüthy et al., 2002), and alginate biosynthesis (Gheorghita et al., 2022), among others. SLR‐proteins are also important for pathogenesis, for instance, EsiB, which contains 11 SLRs contributes to *E. coli* immune evasion by impairing neutrophil activation (Pastorello et al., 2013), while DipA of *Francisella tularensis* is a virulence factor required for intracellular replication (Chong et al., 2013). Moreover, the protein LpnE of *L. pneumophila* plays an important role in host‐cell invasion, phagosome acidification, and the prevention of the LCV fusion to the lysosome (Voth et al., 2019).

## 11 CONCLUDING REMARKS

Alpha‐solenoids represent a significant and diverse subset of STRPs. This review highlights the remarkable structural and functional diversity of alpha‐solenoids, which are crucial across various domains of life. Their essential roles in fundamental cellular processes, such as signaling, apoptosis, and transcriptional regulation, underscore their biological importance and evolutionary relevance.

We have classified alpha‐solenoids into three main folds based on their geometric properties: low and high curvature, and corkscrew. Additionally, we identified eight main subfolds: ANK, HEAT, ARM, TPR, PPR, PUF, TAL, and SLRs. Each subfold showcases unique structural properties and functions, reflecting the versatile nature of alpha‐solenoids. This classification aligns with the evolutionary ensembles of naturally occurring proteins and provides a deeper understanding of how sequence motifs and structural configurations contribute to the specific functions of these proteins.

The association of alpha‐solenoids with various diseases further emphasizes their significance as potential targets for therapeutic research (Llabrés et al., 2020). The increasing availability of accurate structural protein models from advanced machine learning methods, such as AlphaFold (Varadi et al., 2022) and ESMFold (Lin et al., 2023), presents new opportunities and challenges for the large‐scale application of STRP structure‐based predictors (Do Viet et al., 2015; Hirsh et al., 2018; Mozaffari et al., 2024; Qiu et al., 2024). A key resource in this field is RepeatsDB, which offers comprehensive and curated information on alpha‐solenoid proteins and other STRPs. RepeatsDB serves as a reference repository for researchers, providing detailed data on the structural classification and insights that facilitate further study on STRPs' potential in therapeutic interventions and protein design.

## AUTHOR CONTRIBUTIONS

**Paula Nazarena Arrías:** Writing – original draft; writing – review and editing; methodology; visualization; data curation; formal analysis. **Zarifa Osmanli:** Writing – original draft; writing – review and editing; visualization; methodology; formal analysis; data curation. **Estefanía Peralta:** Data curation. **Patricio Manuel Chinestrad:** Data curation. **Alexander Miguel Monzon:** Writing – review and editing; project administration; supervision. **Silvio C. E. Tosatto:** Writing – review and editing; project administration; resources; supervision.

## CONFLICT OF INTEREST STATEMENT

The authors declare that there are no conflicts of interest.

## Supporting information


**Table S1.** Supporting Information.

## Data Availability

All data used in this review is available in publicly accessible repositories.
