# Supplementary material for: Diversity and structural‐functional insights of alpha‐solenoid proteins
Source: Protein Sci. 2024 Oct 28;33(11):e5189. doi: 10.1002/pro.5189 (PMC11514114; doi:10.1002/pro.5189)
Supplement: Supplementary file 1 — Table S1. Supporting Information. [file PRO-33-e5189-s001.pdf]

Supporting Information

Diversity and structural-functional insights of alpha-solenoid proteins

Paula Nazarena Arrías[1,2†], Zarifa Osmanli[1†], Estefanía Peralta[3], Patricio Manuel Chinestrad[4], Alexander Miguel Monzon[5], Silvio C.E. Tosatto[1,6*]

† These authors contributed equally to this work.

* To whom correspondence should be addressed: silvio.tosatto@unipd.it

[1] Department of Biomedical Sciences, University of Padova, via U. Bassi 58/b, 35121 Padova, Italy

[2] Department of Protein Science, KTH Royal Institute of Technology, Stockholm SE-10691, Sweden

[3] Laboratorio de Investigación y Desarrollo de Bioactivos (LIDeB), Departamento de Ciencias Biológicas, Facultad de Ciencias Exactas, Universidad Nacional de La Plata, B1900ADU La Plata, Buenos Aires, Argentina.

[4] Laboratorio de Farmacología Molecular, Universidad Nacional de Quilmes, Roque Saenz Peña 352, 1876 Bernal, Buenos Aires, Argentina

[5] Department of Information Engineering, University of Padova, via Giovanni Gradenigo 6/B, 35131 Padova, Italy

[6] Institute of Biomembranes, Bioenergetics and Molecular Biotechnologies, National Research Council (CNR-IBIOM), Bari, Italy

Keywords: alpha–solenoid subfolds, repeat evolution, protein sequence logo, repeat structure, amino acid conservation

Abbreviation	Name	Interpro repeat	GO terms associated with the Interpro repeat	Interpro homologous superfamily	Pfam	Pfam clan	CATH ID	Structural unit	Representative structure (PDB)
ANK	Ankyrin	IPR002110	GO:0005515	IPR036770	PF12796<br>PF13606<br>PF13857<br>PF13637<br>PF00023	CL0465	1.25.40.20	α/α/big loop<br>or<br>α/α/beta-hairpin	1N11_A
TPR	Tetratricopeptide	IPR019734	GO:0005515	IPR011990	PF13174<br>PF13181<br>PF13176<br>PF00515	CL0020	1.25.40.10	α/α	4Y6W_A
HEAT	HEAT	IPR000357	GO:0005515	IPR011989<br>IPR016024	PF02985	CL0020	1.25.10.10	α/α	4I5N_D
PUF	Pumilio	IPR001313	GO:0003723	IPR011989<br>IPR016024	PF00806<br>PF22493	CL0020	1.25.10.10	α/α/α	3V71_A
PPR	Pentatricopeptide	IPR002885	N/A	IPR011990	PF12854<br>PF13041<br>PF13812<br>PF01535	CL0020	1.25.40.10	α/α	4M57_A
ARM	Armadillo	IPR000225	GO:0005515	IPR011989<br>IPR016024	PF00514	CL0020	1.25.10.10	α/α/α	4DJS_A
TAL	TAL effector repeat	IPR005042	N/A	N/A	PF03377	CL0020	N/A	α/α	3UGM_A
Sel1	Sel1-like repeats	IPR006597	N/A	IPR011990	PF08238	CL0020	1.25.40.10	α/α	5B26_A

Table S1 – Alpha-solenoid subfolds. Database identifiers, representative structures, GO terms correspond to protein and DNA/RNA binding (UniProtKB accessed on 17 june 2024).
